# Navigating the Blood–Brain Barrier: Challenges and Therapeutic Strategies in Breast Cancer Brain Metastases

**DOI:** 10.3390/ijms241512034

**Published:** 2023-07-27

**Authors:** Lucas E. L. Terceiro, Nnamdi M. Ikeogu, Matheus F. Lima, Chidalu A. Edechi, Barbara E. Nickel, Gabor Fischer, Etienne Leygue, Kirk J. McManus, Yvonne Myal

**Affiliations:** 1 Department of Pathology and Laboratory Medicine, Max Rady College of Medicine, University of Manitoba, Winnipeg, MB R3E 3P5, Canada; evangel4@myumanitoba.ca (L.E.L.T.); chidalu.edechi@mail.utoronto.ca (C.A.E.); barbara.nickel@umanitoba.ca (B.E.N.); gabor.fischer@umanitoba.ca (G.F.); 2 Department of Immunology, Max Rady College of Medicine, University of Manitoba, Winnipeg, MB R3E 0T5, Canada; ikeogun@myumanitoba.ca; 3 Department of Physiology and Pathophysiology, CancerCare Manitoba Research Institute, University of Manitoba, Winnipeg, MB R3E 0V9, Canada; matheus.fabiaodelima@umanitoba.ca; 4 Department of Biochemistry and Medical Genetics, Max Rady College of Medicine, University of Manitoba, Winnipeg, MB R3E 0T5, Canada; etienne.leygue@umanitoba.ca (E.L.); kirk.mcmanus@umanitoba.ca (K.J.M.); 5 Paul Albrechtsen Research Institute, CancerCare Manitoba, Winnipeg, MB R3E 0V9, Canada

**Keywords:** blood–tumor barrier, pathogenesis, immunotherapy, therapeutic advantages

## Abstract

Breast cancer (BC) is the most common cancer in women, with metastatic BC being responsible for the highest number of deaths. A frequent site for BC metastasis is the brain. Brain metastasis derived from BC involves the cooperation of multiple genetic, epigenetic, angiogenic, and tumor–stroma interactions. Most of these interactions provide a unique opportunity for development of new therapeutic targets. Potentially targetable signaling pathways are Notch, Wnt, and the epidermal growth factor receptors signaling pathways, all of which are linked to driving BC brain metastasis (BCBM). However, a major challenge in treating brain metastasis remains the blood–brain barrier (BBB). This barrier restricts the access of unwanted molecules, cells, and targeted therapies to the brain parenchyma. Moreover, current therapies to treat brain metastases, such as stereotactic radiosurgery and whole-brain radiotherapy, have limited efficacy. Promising new drugs like phosphatase and kinase modulators, as well as BBB disruptors and immunotherapeutic strategies, have shown the potential to ease the disease in preclinical studies, but remain limited by multiple resistance mechanisms. This review summarizes some of the current understanding of the mechanisms involved in BC brain metastasis and highlights current challenges as well as opportunities in strategic designs of potentially successful future therapies.

## 1. Introduction

Breast cancer (BC) has recently surpassed lung cancer to become the most frequently diagnosed cancer among women in the world [1]. Furthermore, breast cancer represents the second most frequent type of cancer that causes brain metastasis after lung cancer, affecting approximately 10–16% of patients [2]. According to recent statistics from the World Health Organization (WHO), in 2020 alone, BC was the primary cause of cancer-related deaths in women, with over half a million individuals succumbing to this disease [3]. Poor nutrition and lack of physical activity contribute to the greater incidence of BC in developed countries, while the higher mortality rate commonly found in developing countries is most often associated with missed or late diagnosis, lower screening frequencies, and limited therapeutic options [4,5]. Beyond these external factors, metastatic disease (the spread of cancer from the primary site) is an intrinsic event in tumor biology that is responsible for the majority of BC deaths worldwide [6].

BC initially spreads to neighboring lymph nodes that drain the breast, then progresses to distal organs; in particular, the bone, liver, lung, and brain [7]. The preferred metastatic site for BC cells is dependent on factors such as the BC subtype, intrinsic tumor biology, tumor microenvironment, and clonal evolution (acquisition of gene mutations and cellular adaptations that drive metastasis) [8]. An increased risk of brain metastasis is also associated with additional factors such as tumor size, grade stage, lymph node status, age, and the presence of proliferation marker Ki67 [9].

Because specific tumor characteristics can vary greatly from one patient to another [8], clinicians have adopted the presence or absence of the now well-characterized markers as a guide to anticipate the behavior of the lesions and to determine the best course of treatment. Histological features as well as established cell surface markers (progesterone receptor—PR, estrogen receptor—ER, and the human epidermal growth factor receptor 2—HER2), genomic markers (Phosphatidylinositol-4,5-bisphosphate 3-kinase catalytic subunit alpha—PIK3CA, breast cancer gene 1—BRCA1, breast cancer gene 2—BRCA2), proliferation marker (Ki67), and immunomarkers (tumor-infiltrating lymphocytes, programmed death-ligand 1—PD-L1) are routinely used in the clinic for diagnosis [10]. However, the standard approach to the treatment of BC mostly relies on the expression of the molecular markers ER, PR, and HER2. The presence or absence of these markers has enabled the development of targeted therapies, resulting in treatments that are more effective.

### Triple Negative Breast Cancer (TNBC)

The absence of all three molecular markers is referred to as triple negative BC (TNBC) [11]. TNBC is typically a highly metastatic subtype of BC that is difficult to treat, as it often develops resistance to current forms of chemotherapy [12]. In addition to being highly metastatic, TNBC has also been observed to cause brain metastasis more frequently compared with other BC subtypes [13]. The reasons for the increased numbers of TNBC brain metastases are not yet fully understood; however, they might be associated with the aggressive nature of TNBC and its natural ability to invade distant tissues, including the brain.

## 2. Brain Metastasis and Breast Cancer Subtypes

The incidence rates for breast cancer brain metastasis vary according to the breast cancer subtype. HER2-positive and TNBC subtypes are more frequently found causing metastasis to the brain [14]. Approximately 50% of brain metastases attributed to BC are found in patients diagnosed with the TNBC subtype, 33% with the HER2-positive subtype, and 14% with the luminal subtype (Table 1) [13,15,16]. Although most brain metastases occur during the advanced stages of BC, the TNBC subtype progresses very quickly to the brain during the early disease stages. A recent study determined that patients with TNBC had only 22 months between BC detection and brain metastasis, compared with 30 months for HER2-positive BC and 63.5 months for the luminal BC subtype [17,18]. Additional studies reveal that patients with TNBC brain metastasis exhibit the shortest overall survival rate when compared with patients who developed brain metastases from any other BC subtype [16,19,20].

Further studies also show that patients whose BC has metastasized to the brain had poorer outcomes compared with patients who displayed metastatic spread to other sites [21]. Those with brain metastases may also present with neurological comorbidities as well as associated downstream effects, ultimately resulting in a significant increase in mortality rates [22]. The National Cancer Institute′s (NCI) Surveillance, Epidemiology, and End Results (SEER) database estimates that the proportion of metastatic BC patients presenting with brain metastasis is over 7% [23]. Clinical studies show that some patients with brain metastases due to aggressive BC subtypes such as TNBC have a median survival of less than a year [24]. While treatment approaches for BC metastasis to the brain depend on the extent of the disease, general regimens include surgery, radiotherapy (including stereotactic radiosurgery), and chemotherapy [25].

The development of brain metastases is unique in that the cancer cells must cross and modify the blood–brain barrier (BBB) to facilitate invasion into the brain. This very same BBB that restricts cancer cells from crossing into the brain, also limits the access of treatment options to the brain. Thus, chemotherapy, targeted therapy, and hormonal therapy are often unsuccessful due to their inability to cross the BBB [9].

## 3. Brain Metastasis: Mechanisms and Pathophysiology

### 3.1. Dissemination of BC Cells to the Brain

The metastatic process is highly complex and poorly understood, including multiple steps such as genetic and epigenetic alterations, angiogenesis, tumor–stroma interactions, intravasation through the basement membrane, survival in the circulation, and extravasation into distal tissues. It begins with the initial detachment of BC cells from the primary tumor and comprises a sequence of critical and orchestrated steps. This involves invasion of the cancer cells through the basement membrane into adjacent tissues, intravasation into the blood vessels/lymphatic system, survival and arrest in the circulatory system, extravasation via trans-endothelial migration into distant tissues, colonization, and the eventual formation of distant metastatic lesions [26,27,28].

The early steps of tissue invasion and intravasation are critical events necessary to the formation of metastatic lesions and involve the activation of epithelial-to-mesenchymal transition (EMT), extracellular matrix remodeling, and induction of angiogenesis. To intravasate into the circulation, BC cells must break down endothelial junction proteins. Perivascular macrophages or interactions between tumor cells and endothelial cells (ECs) facilitate the intravasation process [29].

During extravasation, metastatic cells present in the circulation cross through the cell junctions between the distant endothelial cells under the support of specific factors, where they can remain dormant. Eventually, surviving metastatic cells escape the dormant stage to form micro-metastatic foci along blood vessels and multiply, interacting with local host cells. One of the primary metastatic sites for a small number of BCs, especially the TNBC subtype, is the brain. However, here, the BC cells are confronted with the formidable challenge of crossing the BBB [28,30].

### 3.2. The Blood–Brain Barrier

**Figure 1:**
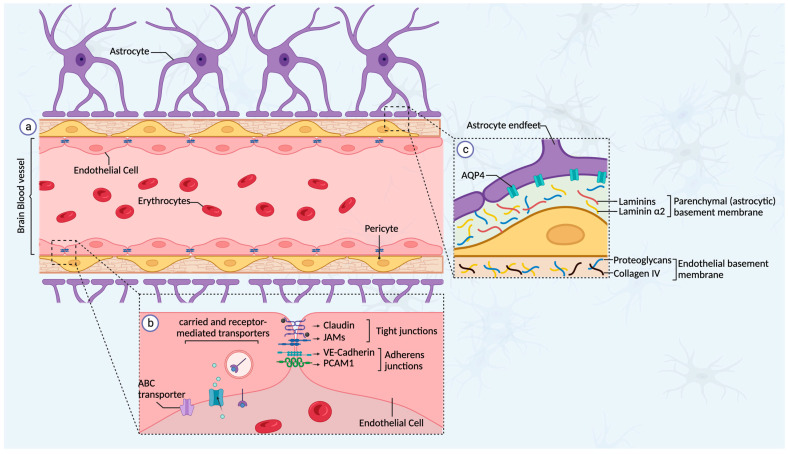
Schematic representation of the blood–brain barrier (BBB). The BBB is a specialized structure comprising endothelial cells (ECs), pericytes, basement membrane, and astrocytes (**a**). The ECs are anchored together by tight-junction (claudins, JAMs) and adherent-junction (VE-cadherin, PCAM1) proteins, thereby limiting any paracellular transport into the brain. The transport of soluble factors into the brain parenchyma is facilitated by carrier-mediated or receptor-mediated transporters (**b**). Pericytes, which surround the blood vessel, also play an important role in regulating BBB permeability by modulating the formation of tight junctions in ECs as well as by secreting the components of the endothelial basement membrane with the support of the ECs. The astrocytes also play a role in supporting the maintenance of the BBB barrier by secreting the components of a second layer of basement membrane (parenchymal basement membrane) and by connecting their endfeet to surround the BBB (**c**).

Metastasis to the brain is a process that, in most cases, is the result of a much longer latency following the initial detection of the primary breast tumor and requires specific cellular adaptations and interactions [31]. The delayed latency in brain metastasis is primarily attributed to the presence of the BBB, a highly specialized structure in the brain microenvironment that limits the access of unwanted molecules or cells into the brain parenchyma [32]. The BBB consists of a protective network comprising polarized ECs connected by adherent and tight junctions, endothelial and parenchymal basement membranes, pericytes, astrocytes (endfeet), and microglia (Figure 1). Not only does the BBB control the permeability of the brain microenvironment to macromolecules, but it is also involved in transmitting signals and maintaining the homeostasis of the nervous system [33]. Microvascular ECs in the brain are crucial to maintaining and ensuring the integrity of the BBB. They constitute the first layer of the BBB and are polarized in structure, with luminal and abluminal surfaces having unique biochemical and functional features [34]. An important feature of the ECs in the BBB is their specific expression of transmembrane proteins, such as occludins (zonula occludens 1 and 2), claudins, JAMs (junctional-adhesion molecules), and accessory proteins that constitute the tight junctions as well as proteins that constitute the adherent junctions (VE-Cadherin, PCAM1, and catenins) [33]. Thus, the entry of substances through the endothelial cells is tightly regulated, and paracellular transport (between ECs) is limited by the expression of proteins that constitute these junctions. The formation of tight and adherent junctions between ECs stabilizes the BBB integrity and creates a non-fenestrated vasculature that prevents unwanted molecules from entering the brain [35]. Consequently, ECs express a variety of uptake and efflux transporters, which play a crucial role in maintaining the brain homeostasis. The transport of soluble factors such as carbohydrates, amino acids, and hormones uses carrier-mediated transporters; however, other substances, such as peptides, transferrin, or growth factors use receptor-mediated transporters [36]. Additionally, the transport of substances from the brain parenchyma or endothelium to the bloodstream is mediated by efflux pumps, such as the ATP-binding cassette family (ABC), that export metabolites and most anticancer drugs [37,38].

Pericytes are found at regular intervals along the capillary walls and play a crucial role in the formation of the BBB by attaching to and creating tight junctions with the ECs. These cells have a direct influence on BBB permeability by regulating the formation of ECs’ tight junctions and adherent junctions [39,40]. In addition, astrocytes, another group of cells, surround the BBB with their endfeet connected to the basement membrane via junctional molecules (i.e., dystroglycan) and channels (i.e., aquaporin 4/AQP4, which helps maintain water balance in the brain). Astrocytes have multiple functions in the regulation of the BBB [41]. They aid in controlling cerebral blood flow by responding to neuronal perturbations via Ca^2+^ signaling. Additionally, astrocytes directly influence vascular growth and proliferation through angiotensin 1 (ANG-1) and vascular endothelial growth factor (VEGF) secretion [33,41,42,43]. The resident immune cells of the brain, known as microglia, have the unique ability to modulate both pro- and anti-inflammatory responses through the expression of either M1 or M2 phenotypes and the release of a variety of molecular cytokines [44,45].

The BBB consists of two basement membranes: an inner endothelial membrane, deposited by ECs and pericytes, and an outer parenchymal membrane, secreted by astrocytes. Rather than just being a solid layer of tissue, the basement membrane acts as a reservoir for secreted signaling proteins and a barrier to incoming unwanted molecules and cells [41].

The specific mechanism by which BC cells cross the BBB remains unclear. However, it has been reported that expression of α2,6-sialyltransferase (ST6GALNAC5) cyclo-oxygenase (COX-2), and the epidermal growth factor receptor (EGFR) ligand HBGF, mediate the passage of BC cells across the BBB. Interestingly, ST6GALNAC5 has been identified as a distinct mediator that plays a role in promoting metastasis to the brain rather than any other organs [46]. Chemokines and their specific receptors are demonstrated to be other factors involved in the migration of BC through the BBB. The stromal-cell-derived factor 1α chemokine (SDF-1α or CXCL12) and its receptor (CXCR4) have been reported to play several roles in BCBM, such as homing, cell motility, and metastasis progression. The CXCL12/CXCR4 signaling pathway plays a crucial role in facilitating the migration of BC cells through the BBB. As well, the expression of CXCL12 by BC cells induces instability in the blood vessels, and it was found to be significantly more abundant in BC cells compared with normal tissues [47,48]. Additionally, among the various chemokine receptors expressed in cancer, CXCR4 is one of those most frequently found at the metastatic site. The activation of CXCL12/CXCR4 has been shown to enhance adhesion and activate trans-endothelial migration by activating PI3K/Akt [48,49]. Altogether, the CXCL12/CXCR4 signaling axis appears to play an important role in facilitating BC cell migration through the BBB and promoting the formation of brain metastasis.

Additionally, extracellular vesicles (EVs) play an important role in facilitating the passage of BC cells across the BBB. Metastatic breast cancer cells can secrete EVs that facilitate the BC cells’ disruption of the BBB. It has been reported that breast cancer cells can secrete small EVs containing microRNA miR-105, which can reduce the expression of ZO-1 (zonula occludens 1) in endothelial cells, resulting in the disruption of the intercellular junctions [50]. More recent studies have now demonstrated that BC cells can transfer the microRNA miR-181c to endothelial cells with small EVs, resulting in the decrease in actin dynamics, which leads to the accumulation of tight-junction proteins in the cytoplasm [51].

### 3.3. The Blood–Tumor Barrier (BTB)

Upon initial metastatic colonization, newly “seeded” tumor cells residing in the brain vasculature activate the process of neo-angiogenesis and microenvironment remodeling to facilitate tumor regrowth and invasion. This results in the formation of a blood–tumor barrier (BTB), a newly established neurovascular-tumor unit with distinct physiological properties when compared with the intact BBB [41].

**Figure 2:**
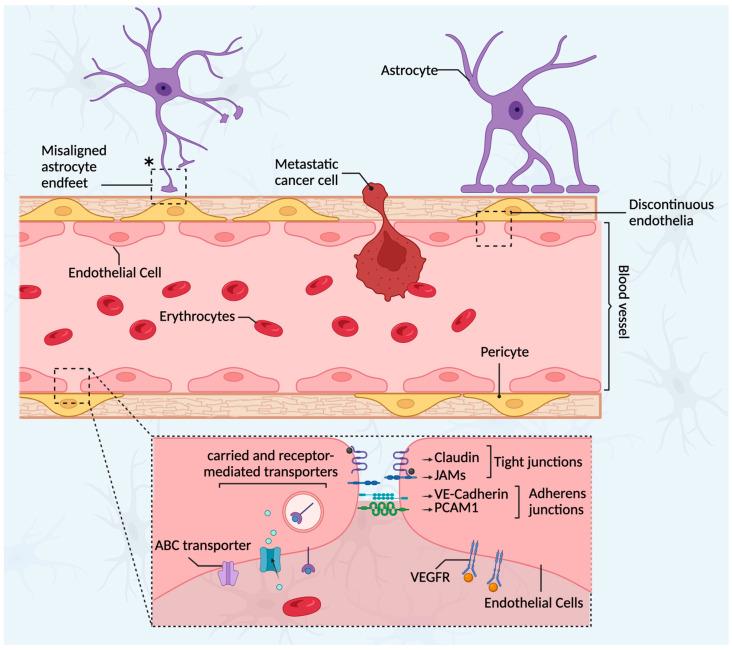
Schematic representation of the brain–tumor barrier (BTB). After initial metastatic colonization in the brain, BC cells induce the process of neo-angiogenesis, resulting in the formation of a new neurovascular-tumor unit known as the BTB. The BTB is not selective when compared with the intact BBB, mainly due to the loss of tight junctions between endothelial cells, which results in a discontinuous endothelium along the blood vessel that allows easy access of many cells, such as cancer and immune cells, to the brain parenchyma. Additionally, the decreasing and the misaligning of the astrocyte endfeet around the blood vessels contribute to the increase in permeability of the BTB (*). To further boost oxygen supply to the brain, endothelial cells stimulate angiogenesis by increasing the expression of VEGFR.

The BTB is inherently “leaky” due to the lack of tight junctions and astrocytic–endothelial cell contacts, resulting in a fenestra and discontinuous endothelia, which contribute to increased permeability (Figure 2) [30]. As metastatic lesions begin to outgrow their oxygen supply, angiogenesis occurs, mainly driven by VEGF [52]. The dynamic angiogenesis that occurs during brain metastatic progression is very heterogeneous among lesions, such that this is thought to be one of the main contributing factors to the heterogeneity observed in tumor permeability to chemotherapy [53].

Astrocytes, pericytes, and microglia confer additional cellular and molecular properties to the BTB. Astrocytes support and protect neuronal cells from damage and apoptosis by secreting inflammatory cytokines such as tumor necrosis factor alpha (TNF-α), interleukin 1 (IL-1), and interleukin 6 (IL-6) [54]. However, the constant release of these cytokines stimulates tumor proliferation and survival. As well, astrocytes release exosomes containing miRNA-19a, which induces loss of the phosphatase and tensin homolog (PTEN) to promote outgrowth and invasion of tumor cells within the brain [54,55]. Microglia in the brain tumor microenvironment also secrete multiple growth factors and cytokines, such as transforming growth factor beta (TGFβ), TNFα, IL1, IL6, VEGF, epidermal growth factor (EGF), as well as metalloproteinases, which further promote tumor proliferation and invasion in addition to supporting angiogenesis [44,45,56]. Microglia cell populations also support metastatic colonization through the wingless-related integration site (Wnt) pathway. Additionally, subpopulations of desmin-positive pericytes are found in high numbers in brain metastases, and their presence is associated with an increased permeability of the BTB [57,58].

Altogether, the distinct physical and molecular properties of the BTB add to the complexity of treating breast cancer brain metastases (BCBMs). Studies using preclinical animal models of BCBMs have revealed that despite the fact that varying levels of BBB disruption are observed, the buildup of chemotherapeutic agents remained restricted, resulting in decreased apoptosis and cytotoxicity in almost 90% of metastatic lesions [58,59]. Such observations have led to the development of techniques targeting disruption of the BBB to improve drug delivery to brain tumor tissues.

### 3.4. The Brain Tumor Microenvironment Cellular Composition

**Figure 3:**
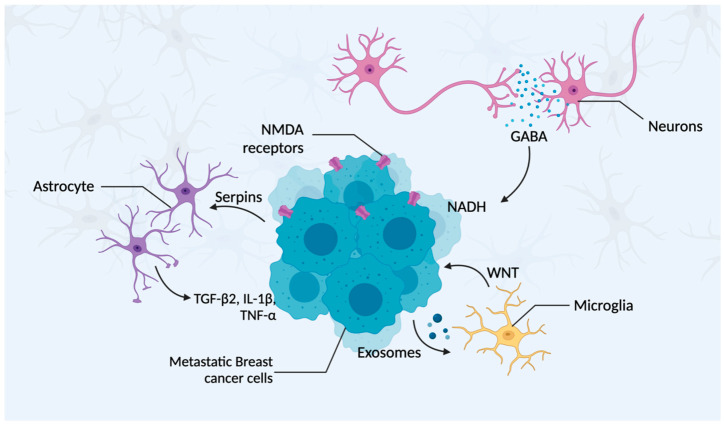
Schematic representation of the established interactions between metastatic BC and host stroma cells. Metastatic BC cells in the brain can express high levels of serpins to prevent the astrocyte metastasis-suppressive effect and can also stimulate these astrocytes to secrete TGF-β, IL-1β, and TNF-α, leading to tumor cell expansion. These BC cells can also secrete exosomes that stimulate microglia to support tumor progression through Wnt signaling and microglia polarization to an M2 phenotype. Furthermore, BC cells can exploit neurotransmitters secreted by neurons (e.g., GABA), as bio-precursors for the generation of NADH, further promoting tumor cell proliferation.

For BC cells to survive and expand at the metastatic site, they need to adapt to the new microenvironment and develop interactions with surrounding host stroma cells (Figure 3). In the brain, the microenvironment primarily comprises neurons, astrocytes, and microglia [41], which are described in greater detail below.

#### 3.4.1. Neurons

Neurons are the essential functional component of the central nervous system (CNS). Recent studies have demonstrated that within the brain microenvironment, BC cells adopt a brain-like phenotype, exhibiting characteristics similar to native neurons, with elevated GABA (γ-aminobutyric acid) receptor and transporter levels. These attributes provide a proliferative advantage for the BCBM cells, as the augmented GABA uptake and metabolism promote NADH formation, subsequently boosting adenosine triphosphate production [60]. Studies conducted by Zeng et al. (2019) showed that BC cells are capable of glutamate uptake, which can then activate N-methyl-D-aspartate (NMDA) receptors. This activation is essential for metastatic colonization within the brain and is correlated with an unfavorable prognosis [61].

#### 3.4.2. Astrocytes

Astrocytes are considered the most abundant glial cells present in the brain parenchyma, and they exhibit a crucial role in sustaining the BBB. These cells surround the brain micro-blood vessels and neuronal processes; express functional proteins, such as dystroglycans, dystrophin, and AQP4; and secrete laminin α1 and α2, which play critical roles in preserving the BBB [35,62]. It has been suggested that during the initial process of brain colonization by BC cells, astrocytes target and attack the newly seeded metastatic cells as a defense mechanism. As well, reactive brain-stroma-derived plasmin cleaves the Fas ligand (FasL) present on the surface of the astrocytes, facilitating interaction with its receptor, Fas, and consequently inducing apoptosis in the tumor cells. However, plasminogen activator (PA) inhibitory serpins, which are highly expressed in BCBM cells, can disrupt this process [54,63]. Gong et al. (2019) demonstrated that TNBC cells exposed to astrocyte-conditioned medium (ACM) have a higher tendency to form brain metastases, with upregulation of angiopoietin-like 4 (ANGPTL4) being a key factor [64]. Knockdown of ANGPTL4 in TNBC cells decreased the ACM-induced tumor cell metastatic growth in the brain and improved survival. This study also determined that astrocytes produce transforming growth factor-beta 2 (TGF-β2), which is responsible in part for ANGPTL4 upregulation via suppressor of mothers against decapentaplegic (SMAD) signaling. They also showed that tumor cells communicate with astrocytes, thereby increasing TGF-β2 expression through interleukin-1 beta (IL-1β) and tumor necrosis factor alpha (TNF-α) [64,65]. Altogether, these findings suggest that TNBC cells interact with astrocytes, facilitating brain metastasis through a TGF-β2/ANGPTL4 axis.

BC cells that survive the brain microenvironment can exploit the astrocytes through communication via gap junctions composed of connexin 43 (Cx43) and protocadherin 7 (PCDH7). This interaction provides the BC cells with advantages in growth, chemoresistance, stemness, and autophagy [66]. The transfer of the second messenger cGAMP from tumor cells to astrocytes leads to the increased secretion of interferon-α (IFN-α) and tumor necrosis factor-α (TNF-α) by the astrocytes. This, in turn, activates the signal transducer and activator of transcription 1 (STAT1) and nuclear factor κB (NF-κB) pathways in the brain metastatic cells to promote tumor growth and chemoresistance [67].

Astrocytes also can release polyunsaturated fatty acids, which can then trigger the activation of peroxisome proliferator-activated receptor γ (PPARγ) in BCBM cells, thereby leading to increased proliferation. Furthermore, astrocytes can modify the structure of the BCBM cells through cytoskeleton remodeling, improving actin stress fiber organization, and promoting cell elongation, ultimately enhancing their migratory capacity [68,69].

#### 3.4.3. Microglia

Microglia are a unique group of resident macrophages in the CNS that function to clear cellular debris and actively survey the brain parenchyma. Microglia exhibit two distinct activation states with contrasting roles in brain metastasis [56]. The M1-like phenotype stimulates BBB disruption to enable leukocyte infiltration, while the M2-like phenotype contributes to angiogenesis and immunosuppression. Thus, together the M1 and M2 microglia promote tumor progression [70]. When stimulated by physical contact, microglia produce and accumulate reactive oxygen species (ROS), which trigger apoptosis or other oxidative damage as part of the cellular immune defense. Moreover, the impact of these processes is mitigated by the increased expression of MYC in the BCBM cells, which in turn stimulates the production of glutathione peroxidase 1 (GPX1), an antioxidant enzyme [71,72].

Like other immune cells, microglia also perform cytotoxic functions. However, during brain colonization, metastatic BC cells can secrete high levels of neurotrophin-3 (NT-3), which promotes metastatic growth by reversing EMT to mesenchymal–epithelial transitions (METs), thereby increasing the cellular expression of E-cadherin [73,74]. As well, BC cells can increase the secretion of exosomes containing miRNA-503, resulting in the microglia M2 phenotype polarization and accumulation of immune-suppressive cytokines in the microglia that subsequently inhibit T-cell proliferation [75,76,77].

## 4. Signaling Pathways Involved in BCBM

Cancer stem cells are widely recognized as the primary initiators of tumorigenesis and metastasis. The Notch and Wnt signaling pathways are crucial to maintaining normal stem cell function but have also been implicated in cancer stem cells, with deregulation observed in multiple malignancies, including BC, glioblastomas, and lung cancer [78,79,80]. An expanding collection of evidence shows the importance of the Notch signaling pathway in maintaining the stem-like properties of BC stem cells in distinct microenvironments [81]. Earlier studies demonstrated that BC cells with a high propensity to metastasize to the brain (MDA-MB-231) exhibit increased activation of the Notch pathway via Notch1 and Jagged-2 (JAG2) [82]. Moreover, studies by Xing et al., 2013, determined that BC cells located in the brain display an elevated expression of interleukin 1β (IL-1β), which stimulates local astrocytes to express Jagged-1 through the NF-κB signaling pathway. This interaction between astrocytes and BC stem cells greatly enhances the activation of the Notch signaling pathways within the cancer cells [83]. Notably, earlier studies demonstrated that silencing Notch1 in MDA-MB-231 cells diminishes the CD44^high^/CD24^low^ phenotype, leading to reduced brain metastasis [84]. Additionally, microglia can contribute to the infiltration and colonization of brain tissue by BC cells by functioning as active transporters and guiding rails in a Wnt-dependent manner [57].

Numerous studies have also explored the clinical and functional significance of EGFR, HER2, HER3, and the associated downstream signaling pathway components, including phosphoinositide 3-kinases (PI3K), serine/threonine kinase (AKT), mammalian target of rapamycin (mTOR), and PTEN in the context of BCBM [85,86,87,88]. The PI3K/AKT/mTOR axis is known to impact BC cell growth, survival, migration, and metabolism and holds a considerable influence in the regulation of CNS metastasis [89,90,91]. Studies by Blazquez et al., 2018, have shown that the PI3K/AKT/mTOR pathway leads to the increased expression of immune-related genes (PD-L1, CSF1, and CSF1R) or cytotoxic T-lymphocyte-associated protein 4 (CTLA4) in microglia or cancer cells within the brain metastasis microenvironment. The expression of these genes and the invasive BCBM cells significantly diminish when a pharmacological inhibitor targeting the PI3K/AKT/mTOR signaling pathway is employed [92].

Alternatively, PTEN is a lipid phosphatase that exhibits a pivotal role in the negative regulation of the PI3K/AKT signaling pathway. Thus, loss of PTEN in neoplastic cells underlies the activation of the PI3K/AKT pathway [93,94]. This was demonstrated by Wikman et al., 2012, who reported that the expression of PTEN was considerably lower in brain metastases than in nonmetastatic primary tumors. The frequency of PTEN gene mutations in the BCBM was significantly higher than that of the primary tumor in the mammary gland [95]. Further studies by the same group found that BC cells with a normal expression of PTEN would lose PTEN expression upon brain metastasis but restore its levels once leaving the brain microenvironment. Interestingly, the modulation of this mechanism was governed by astrocyte-derived microRNAs (miRNAs). As well, the depletion of PTEN in cerebral metastatic cells led to an upregulation of cytokine chemokine (C-C motif) ligand 2 (CCL2), consequently fostering the proliferation of brain metastatic tumor cells [55].

The ERBB family of receptor tyrosine kinases (RTKs) comprises EGFR, alternatively designated as ERBB1 (HER1), ERBB2 (HER2), ERBB3 (HER3), and ERBB4 (HER4) [96]. Members of this family contribute to the regulation of essential cellular functions, such as differentiation, proliferation, angiogenesis, migration, survival, apoptosis, and metabolism, by activating downstream signaling cascades, including PI3K/Akt, Ras/MEK/ERK, Janus-activated kinase/signal transducer and activator of transcription (JAK/STAT), as well as phospholipase Cγ (PLCγ)/PKC [97].

Within the ERBB family, the expression of HER2 and EGFR is frequently increased in various cancer types, including BC [98]. In fact, there is a correlation between HER2 overexpression and the development of brain metastases in BC patients [99]. In vivo studies suggest that increased HER2 expression enhances the expansion of BCBM [100]. The overexpression of HER3, another member of the EGFR family, is also linked to the development of brain metastases in individuals with BC. The increased prevalence of brain metastases in HER2/HER3-positive BC patients is attributed to several factors [101]. There is a significant level of heregulin, the primary ligand of HER2/HER3 heterodimers, in the human brain, and a growing body of evidence suggests that it promotes the trans-endothelial migration of HER2/HER3-positive BC cells across the brain microvascular endothelia. This process is mediated by the activation of intracellular pathways leading to the secretion of MMP-9 [102]. Although the treatment of HER2-positive patients with trastuzumab prolongs the lifespan of the patient, its limited ability to penetrate the BBB may in fact cause the brain to serve as a “sanctuary” site for metastases, allowing brain metastasis to manifest more prominently with time [103].

## 5. Current Treatments and Therapies for BCBM

Despite advances in the early detection and treatment of BCBM, the 5-year overall survival (OS) rate remains lower than 30% [28]. Local stereotactic radiosurgery (SRS) and whole-brain radiotherapy (WBRT) result in early and late neurotoxicity, without any considerable improvement in OS [16,104,105,106]. Standard systemic chemotherapy has been observed to increase OS in BCBM patients compared with no chemotherapy [107,108], but an increase in the rate of metastatic progression has also been reported. However, these reports lack clarification of the direct effect of the systematic approach on BCBM [107,108]. As well, a low treatment efficacy was also observed using immunotherapy alone or associated with other treatment approaches. Once the BCBM evolves multiple resistance mechanisms to evade the immune system, the physical impairment imposed by the BTB and formation of a new tumor microenvironment supports the growth of the metastatic tumor [109,110,111].

### 5.1. Utilization of New Anticancer Drugs

While local interventions remain the most important method of targeting brain metastasis in BC, systemic therapy plays a significant role in the treatment. As well, monoclonal antibodies (trastuzumab and pertuzumab) and tyrosine kinase inhibitors (TKIs; lapatinib, neratinib, tucatinib, and pyrotinib) combined with capecitabine are also frequently utilized. More recently, antibody–drug conjugates (ADCs; trastuzumab emtansine and trastuzumab deruxtecan) have also been employed as a strategy for treating HER2+ BC with brain metastasis [112]. Initial studies primarily focused on TKIs because of their small molecular mass. However, it soon became evident that due to the extensive damage of the BBB attributed to metastasis, the larger ADCs were also able to penetrate the brain parenchyma, thereby providing superior outcomes. In a recent single-arm, phase II clinical trial (TUXEDO-1), trastuzumab deruxtecan exhibited a high intracranial response rate of 73.3% and median progression-free survival (PFS) of 14 months [113]. In the hormone receptor positive/HER2 negative (HR+/HER2−) and the TNBC subset of BC patients with brain metastasis, the overall prognosis and response to therapies is less successful, with fewer studies on potential systemic treatments [114]. Some responses have been documented on the use of aromatase inhibitors and fulvestrant in HR+/HER2− patients, while in TNBC, the anti-angiogenic bevacizumab and the microtubule inhibitor eribulin have shown some CNS activity [115,116]. The list of potential new drugs being tested for brain metastasis includes PARP inhibitors, PI3K inhibitors, ATM inhibitors, and blood–brain barrier disruptors [25].

### 5.2. Utilization of Immunotherapy

Due to the limited brain penetration of drugs administered systemically and the previous belief that brain metastases are poorly immunogenic, patients with brain metastasis were initially excluded from clinical trials involving systemic immunotherapies (ITs) [117]. However, it is currently known that enhancing immune responses against BCBMs does improve the disease outcome [25]. Immunotherapeutic strategies against BCBMs can be classified broadly into two categories: (1) those that enhance immune responses, leading to anti-tumor activity such as T-cell-focused immunotherapies and vaccinations, and (2) those that inhibit immunosuppression, thereby removing the brakes on anti-tumor immunity (e.g., immune checkpoint inhibitors, ICIs; tumor-associated macrophages, TAMs; and microglia-targeted therapies). Currently, several immunotherapies have been employed in treating BCBMs, some of which are highlighted below.

#### 5.2.1. T-cell-Focused Immunotherapies

The immunotherapeutic strategies not directed against immunosuppression, mostly are directed toward improving the anti-tumor responses of T cells [118]. Adoptive cell therapy (ACT) involves the expansion of T-cell-receptor (TCR)-transduced lymphocytes or autologous tumor-infiltrating lymphocytes (TILs), which are later transferred back into the patient in the presence or absence of lymphodepletion and/or concurrent BC. Eight infusions of polyclonal activated T cells, transduced with anti-CD3 and anti-HER2 bispecific antibodies (HER2Bi) [119], resulted in anti-tumor responses [120]. Furthermore, another form of ACT that has yielded promising results in the management of solid tumors is the adoptive transfer of chimeric antigen receptor (CAR)-engineered T cells [121]. Priceman et al., 2018, demonstrated that HER2-targeted CAR (HER2-CAR) T cells containing tumor necrosis factor ligand superfamily member 9 (4–1BB) intracellular co-stimulatory domains suppressed T-cell exhaustion as well as enhanced proliferative capacity compared with those with the CD28 domain for co-stimulation [121].

#### 5.2.2. Vaccinations

Most cancer vaccinations involve dendritic cells (DCs), which are antigen-presenting cells (APCs) that capture and present antigens to T cells for activation [122]. Because BC is now considered an immunogenic disease, various BC tumor-associated antigens (TAgs), including HER2 and mucin 1 (Muc1), are currently being explored as potential vaccines for patients with extracranial BC tumors [123,124]. Several ongoing clinical trials are investigating the potential of DC vaccines as potential therapeutic agents against BCBMs in patients with BC (NCT02808416, NCT01782274), including a phase I trial investigating the autologous, tumor-lysate-pulsed DC vaccine DCVax-Direct [125].

#### 5.2.3. Immune Checkpoint Inhibitors (ICIs) Targeted Therapies

Immune checkpoint blockade involves the use of ICIs that target molecules such as programmed cell death protein 1 (PD-1) and cytotoxic T-lymphocyte-associated protein 4 (CTLA-4) inhibitory receptors, expressed on T cells, and their ligands (PD-L1 and PD-L2), expressed on tumor cells and stromal cells, to avert immunosuppression [126]. To increase their endurance in a patient, cancer cells may co-opt these inhibitory signaling pathways to evade recognition and elimination by host T cells, thereby counteracting host anti-tumor immune responses. [126]. Currently, some available ICIs include monoclonal antibodies against PD-L1 (durvalumab, avelumab, and atezolizumab), PD-1 (pembrolizumab, nivolumab, and cemiplimab), and CTLA-4 (ipilimumab and tremelimumab) [127,128]. Combination therapies involving these ICIs have been reported to increase ICI efficacy. For instance, a combination of nivolumab and ipilimumab enhanced the response rates in some melanoma patients (approx. 50–55%) [127,128] compared with ipilimumab alone, with response rates of 16–25% [129]. One drawback to the use of combinatorial ICI therapy is the presence of adverse effects in 96–97% of the patients that received dual ICI therapy compared with 68% in monotherapy trials with nivolumab [130].

#### 5.2.4. Tumor-Associated Macrophages (TAMs) and Microglia-Targeted Therapies

In addition to ICI, strategies targeting specific cells are also being investigated as therapies for treating BCBMs. For instance, the PI3K pathway is reported to be activated in over 70% of BCBMs [87,131], and it plays a critical role in the metastasis-promoting capacity of TAMs by enhancing the expression of immunosuppressive genes like PD-L1 and the colony-stimulating factor (CSF) 1 receptor (CSF1R) [132]. The use of BKM120 (buparlisib) to inhibit PI3K in infiltrating TAMs [133] led to the repolarization of TAMs to a more anti-tumor phenotype, resulting in a reduction in BC infiltration into the brain parenchyma tissue [132]. However, a phase II clinical trial of BKM120 in patients with metastatic TNBC recently showed that BKM120 alone may not be sufficient in preventing metastasis in this BC subtype [134]. Nevertheless, there is currently an ongoing phase II clinical trial (NCT02000882) of BKM120 in combination with chemotherapy (capecitabine) to prevent BCBM in patients; the results have not yet been published in a peer-reviewed journal. It is now well recognized that the activation of PI3K signaling pathways leads to downstream activation of the protein kinase B (Akt) as well as the mechanistic target of rapamycin (mTOR) pathways [135]. These pathways have been implicated in the progression of different cancers, including BC [135]. Therefore, various drugs have now been engineered to target some critical components of these pathways in an effort to prevent BCBMs, although only a few of them have made it to clinical trials [135]. Everolimus is an mTOR complex 1 (mTORC1) inhibitor with the ability to penetrate the BBB [135] that was approved for late-stage HER + BC patients in combination with aromatase inhibitors [136]. Currently, everolimus, in combination with vinorelbine and trastuzumab, is in a phase II clinical trial against BCBMs [137]. Furthermore, the CSF1/CSF1R signaling axis (downstream effector of the PI3K pathway) involved in the differentiation and survival of macrophages [138] has been under active investigation against BCBMs. In a murine model, TAM CSF1R signaling promotes the intravasation and invasiveness of BC [139]. Therefore, using murine BC models, studies show that neutralizing the CSF1/CSF1R signaling pathway with anti-CSFR1 antibodies and some small inhibitory molecules leads to reduced tumor growth by suppressing TAM and enhancing CD8^+^ T-cell infiltrations, respectively [140].

### 5.3. Additional Therapies

Current studies have focused on the role of truncated glioma-associated oncogene homolog 1 (tGLI1) as a BCBM driver [141]. tGLI1 is a splicing variant of the oncogenic transcription factor GLI1 with multiple regulation sites in the genome, promoting angiogenesis, invasion, tumor growth, migration, and stemness [141,142,143,144]. The expression of tGLI1 in different tumor types, including BCBM, and its absence in normal tissues, draws attention to its potential as a specific therapeutic target to treat BCBM [141,142,143,144,145].

The FDA (U.S. Food and Drug Administration, Silver Spring, MD, USA) has recently approved the use of ketoconazole (KCZ), an antifungal agent (already in clinical use), for the treatment of BCs that express tGLI1 [146]. In mouse models of BCBM, KCZ was shown to inhibit tumor progression [146], and KCZ-derived compounds exhibited pronounced BTB diffusion while keeping tGLI1 target specificity [146]. Preliminary results from an ongoing phase I clinical trial (NCT03796273) demonstrated that KCZ was able to accumulate in BCBM samples, highlighting its ability to penetrate the BTB [147].

The activation of the PI3K/AKT/mTOR pathway, present in 40–70% of BCBM patients [89,90], confers resistance, adaptability, and survival of BCBM-associated circulating tumor cells (CTCs), promoting the establishment of secondary tumors in the brain [127,148,149]. Targeting the PI3K/AKT/mTOR pathway in orthotopic patient-derived xenografts of HER2+ BC in mice promoted tumor regression and increased survival [150]. In the BCBM orthotopic patient-derived xenografts model, the pan-Akt inhibitor GDC-0068 inhibited tumor growth and increased the OS rate compared with the control mice [89,90]. GDC-0068 is also being investigated for the treatment of gliomas and glioblastomas due to its inherent ability to permeate the BTB [151,152], and it may hold promise as a treatment strategy for BCBM. Additionally, a dual PI3K/mTOR inhibitor, GDC-0084, is currently under investigation for the treatment of BCBM, with both in vitro and in vivo models showing a high efficacy with few side effects [89]. As well, an ongoing clinical trial (NCT03765983) is investigating the use of GDC-0084 combined with trastuzumab to treat BCBM derived from HER2 + BC [153].

## 6. Future Perspectives

The frequency of BCBM has increased over the past two decades, likely due to the substantial improvements in overall survival of advanced BC patients. However, new knowledge of the key players contributing to this disease process, as well as the development of recent technologies, are providing new opportunities and paving the way for the orchestration of a more personalized approach to BC treatment in the clinic.

### 6.1. Addressing the BBB

A crucial step in the spread of BC to the brain is overcoming the BBB. Therefore, it is essential to fully understand how BC cells interact with the BBB to facilitate their entry into the brain. Another group of players and a key component of the BBB are the tight-junction proteins claudins and occludins [35]. Tight-junction proteins play an important role in maintaining the integrity of blood vessels in nonpathological conditions. However, studies show they also exhibit a role in cancer development and possibly metastasis. Our group has focused on broadening our understanding of the role of the tight-junction protein Claudin-1 in BC. We have previously shown that Claudin-1 expression in ER-negative BC correlates with markers of the basal-like phenotype [154] and have observed the possibility of a “claudin high” subset of BCs, suggesting that Claudin-1 may be a multifaceted player in cancer progression [155].

Although Claudin-1 promotes collective migration in human BC cell lines [156] and is downregulated in invasive human BC [157], little is known about its potential role in metastases to the brain. There is accumulating evidence to suggest that it has an impact on the BBB during certain disease states. For example, in a mouse model of stroke, increased Claudin-1 levels were associated with increased permeability of the BBB post stroke [158]. As well, in a mouse model of multiple sclerosis, Claudin-1 is associated with reduced BBB permeability, especially in the chronic course of the disease [159]. In some cancers that metastasize to the brain, Claudin-1 has been implicated to play a role. For instance, in melanoma, there is a report of an interaction of Claudin-1 with brain ECs, resulting in metastatic cells being inhibited from entering the brain [160].

### 6.2. Accessing the Blood–Brain Barrier

Because the BBB limits the entry of medications used to treat metastatic lesions, several methods have been investigated to make the BBB more permeable, including the use of intrathecal and intra-arterial injections as well as radiotherapy. However, further research is still needed to optimize such approaches. The slow progress in immunotherapy for BCBMs has been attributed to limited known targets, limitations for drug delivery, as well as substantial safety concerns. Emerging studies suggest that nanomedicine may be a tool that could be explored to improve the progress of immunotherapy in BC brain metastasis [110]. Nanoparticles, which are small and can be designed to cross the BBB, have been developed to deliver anticancer medications (such as chemotherapy) to the brain [161]. However, there is little evidence to support the use of nanotherapy for the treatment of BCBM clinically [161]. More clinical trials demonstrating the efficacy of nanotherapeutic agents are required before adopting this technology in clinical practice.

### 6.3. Personalized Medicine

Genomic profiling of BCBM samples reveals an altered genomic landscape in BCBM when compared with cells in the primary tumor [162,163]. These key adaptations drive the primary tumor cells to invade the systemic blood circulation and enable them to thrive at distant organs, further establishing secondary tumors [164]. The identification of metastatic drivers in primary tumors and CTCs could serve as predictive and prognostic tools for the treatment of BCBM [164]. Furthermore, detection of metastatic driver genes could reveal potential therapy targets, specifically for BCBM derived from TNBC tumors [165,166]. However, because BCBM tumor biopsies are in most cases impossible to attain, genomic mapping of BCBM cells is unlikely, highlighting the importance of multi-omics screening of CTCs to propose a personalized treatment approach [167].

### 6.4. Targeting the EMT Process

It is now well recognized that EMT is a key metastatic driver in BC [168]. EMT is required for cellular detachment from the primary tumor and the establishment of CTCs that can further colonize distant organs [169,170]. The downregulation of epithelial markers such as claudin, E-cadherin, and epithelial cell adhesion molecule (EpCAM), and the upregulation of mesenchymal markers, vimentin, CD44, and ALDH1A3, are key events during the EMT process [169,170,171,172]. Given the importance of EMT to the metastatic process in BC, only a few studies have explored this therapeutically. Targeting the ALDH1A3 gene using shRNA or using the small inhibitor MF-7 demonstrated impairment of tumor formation in a BCBM human xerograph mouse model [172]. Furthermore, downregulation of ALDH1A3 in glioblastoma cells was able to restore drug sensitivity in temozolomide-resistant cells [173], which suggests its use in different brain tumors. The development of drugs targeting EMT in BC is needed to determine their clinical utility as potential coadjutants in the treatment of BC patients with high risk of brain metastases.

## 7. Conclusions

In this review, we explored recent advances in the research and management of BCBM. Significant progress has been made in several aspects of our understanding of BCBM, including the areas of genomic technologies. Despite these remarkable advancements, it is imperative that researchers continue working to both further our understanding of BCBM and to develop therapeutic approaches to ultimately improve the prognosis and quality of life for patients afflicted with BCBM. Collaborative efforts across multidisciplinary teams will be crucial to drive forward this essential research, developing innovative and personalized treatment modalities for BCBM patients.

## Figures and Tables

**Table 1:** BC subtype relevance to brain metastasis incidence and OS rate.

Subtype	Molecular Marker	Ki-67	Incidence of CNS Metastasis	OS Rate after BCBM ^1^
Luminal A and B	ER+, PR+, HER2−	Low or high	14%	7.1–9.3
HER2-positive	ER+, PR+, HER2+	High	33%	11.5–18.9
TNBC	ER−, PR−, HER2−	High	50%	4.4–4.9

^1^ Numbers are represented in months. CNS: central nervous system; OS: overall survival; BCBM: breast cancer brain metastases [13,15,16].
